# Lipoprotein apheresis: still needed in many patients, or soon a historical practice?

**DOI:** 10.1093/ckj/sfag240

**Published:** 2026-08-04

**Authors:** Volker J J Schettler, Yamac Akgun, Jürgen Floege, Carmine Zoccali

**Affiliations:** Center of Nephrology Göttingen GbR, Göttingen, Germany; University of Miami Miller School of Medicine, Department of Pathology and Laboratory Medicine; Department of Nephrology and Immunology, RWTH Aachen University Hospital, Aachen, Germany; Associazione Ipertensione, Nefrologia, Trapianto Renale (IPNET), Reggio Cal, Italy; Institute of Biology and Molecular Genetics, Ariano Irpino, Italy; Renal Research Institute, NY, USA

**Keywords:** atherosclerosis, coronary heart disease, lipoprotein apheresis, peripheral arterial disease, stroke

## Abstract

Lipoprotein apheresis (LA) has long been used as an ultima ratio therapy for patients with extreme lipid-driven cardiovascular risk. One perspective maintains that, despite major advances in lipid-lowering pharmacology and the emergence of gene-silencing therapies, LA remains indispensable for selected patients with homozygous familial hypercholesterolaemia, severe hypercholesterolaemia refractory to maximal therapy, and isolated lipoprotein(a) [Lp(a)]–mediated progressive cardiovascular disease. An opposing view argues that modern and emerging pharmacological agents—PCSK9 inhibitors, inclisiran, bempedoic acid, evinacumab, and targeted Lp(a)-lowering antisense and small interfering RNA agents—have already rendered LA obsolete in almost all patients, with its remaining niche closing rapidly as outcome data accumulate. This combined manuscript juxtaposes the two positions in a streamlined form and concludes with a balanced conclusion of the future role of LA.

## Graphical Abstract

**Graphical Abstract:**
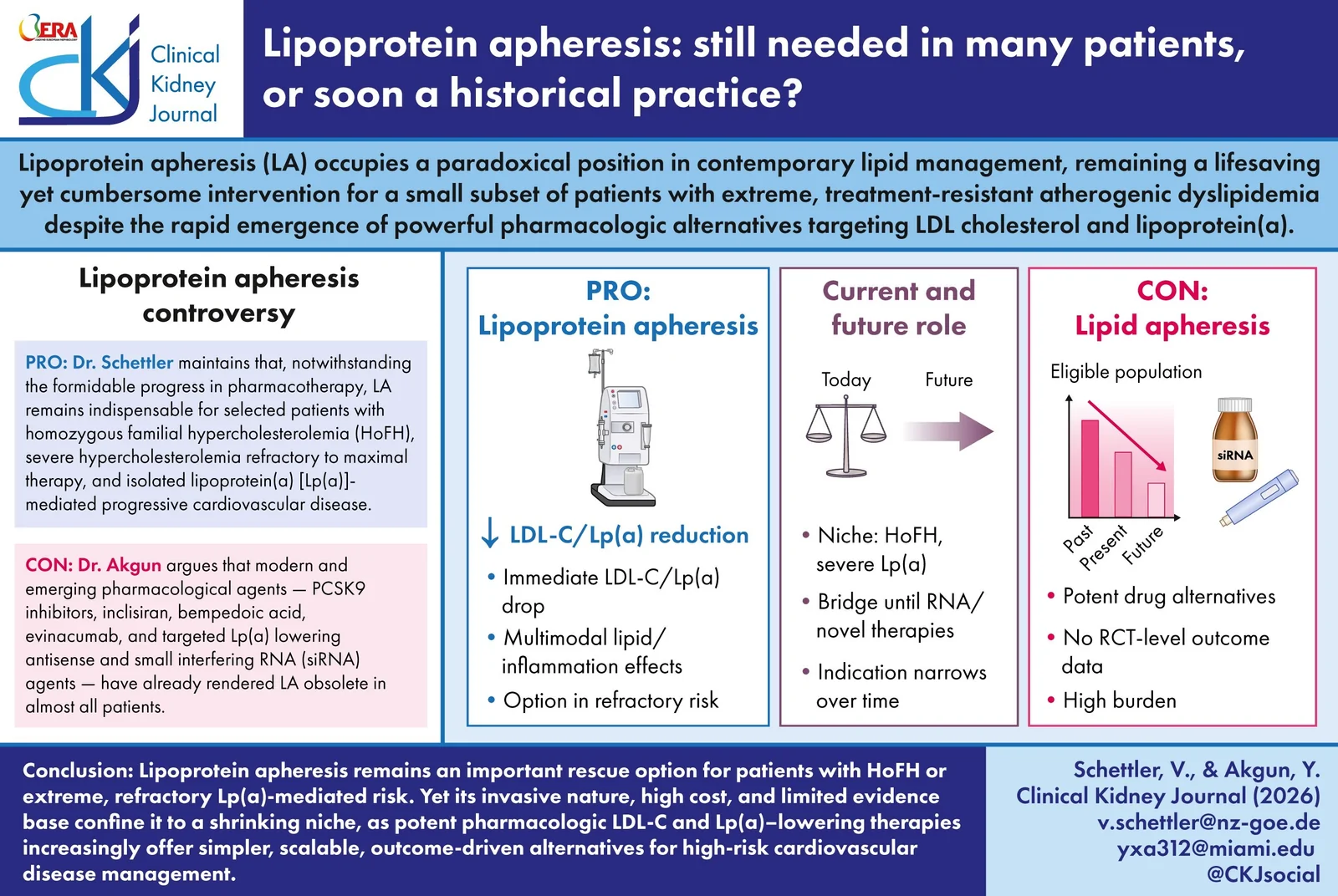

## INTRODUCTION

Atherosclerotic cardiovascular disease (ASCVD) in the context of genetic dyslipidemias such as homozygous familial hypercholesterinaemia (HoFH) and severe heterozygous familial hypercholesterolaemia (HeFH) is associated with a high burden of premature morbidity and mortality [1–3]. Historically, lipoprotein apheresis (LA) emerged as an extracorporeal debulking therapy capable of rapidly removing atherogenic lipoproteins from circulation, when effective pharmacological options were extremely limited. It was initially adopted for conditions characterized by extreme elevations of low-density lipoprotein cholesterol (LDL-C) and, later, for conditions driven by markedly elevated Lp(a).

Over recent decades, the therapeutic landscape has changed profoundly. High-intensity statins, ezetimibe, PCSK9 monoclonal antibodies, inclisiran, bempedoic acid, and evinacumab have provided potent, scalable means of lowering LDL-C. At the same time, antisense oligonucleotides and siRNA agents specifically targeting Lp(a) synthesis are in advanced clinical development. Against this shifting background, the question is no longer whether LA was justified in its historical context, but whether its continued use is justified in contemporary and future practice.

In this controversy, Dr Schettler [4] argues the PRO position that LA remains an indispensable ultima ratio therapy for selected patients at very high cardiovascular risk, including those with HoFH, severe hypercholesterolaemia refractory to maximal lipid-lowering therapy, and isolated Lp(a)-driven progressive cardiovascular disease. In contrast, Dr Akgun [5] presents the CON position, contending that advances in pharmacological lipid modification—particularly PCSK9 inhibition, inclisiran, and emerging Lp(a)-targeted RNA therapeutics—are rapidly shrinking the clinical niche for LA, which is likely to become a historical practice except in rare, extreme phenotypes.

### PRO position: lipoprotein apheresis is still needed in many patients

**Figure 1:**
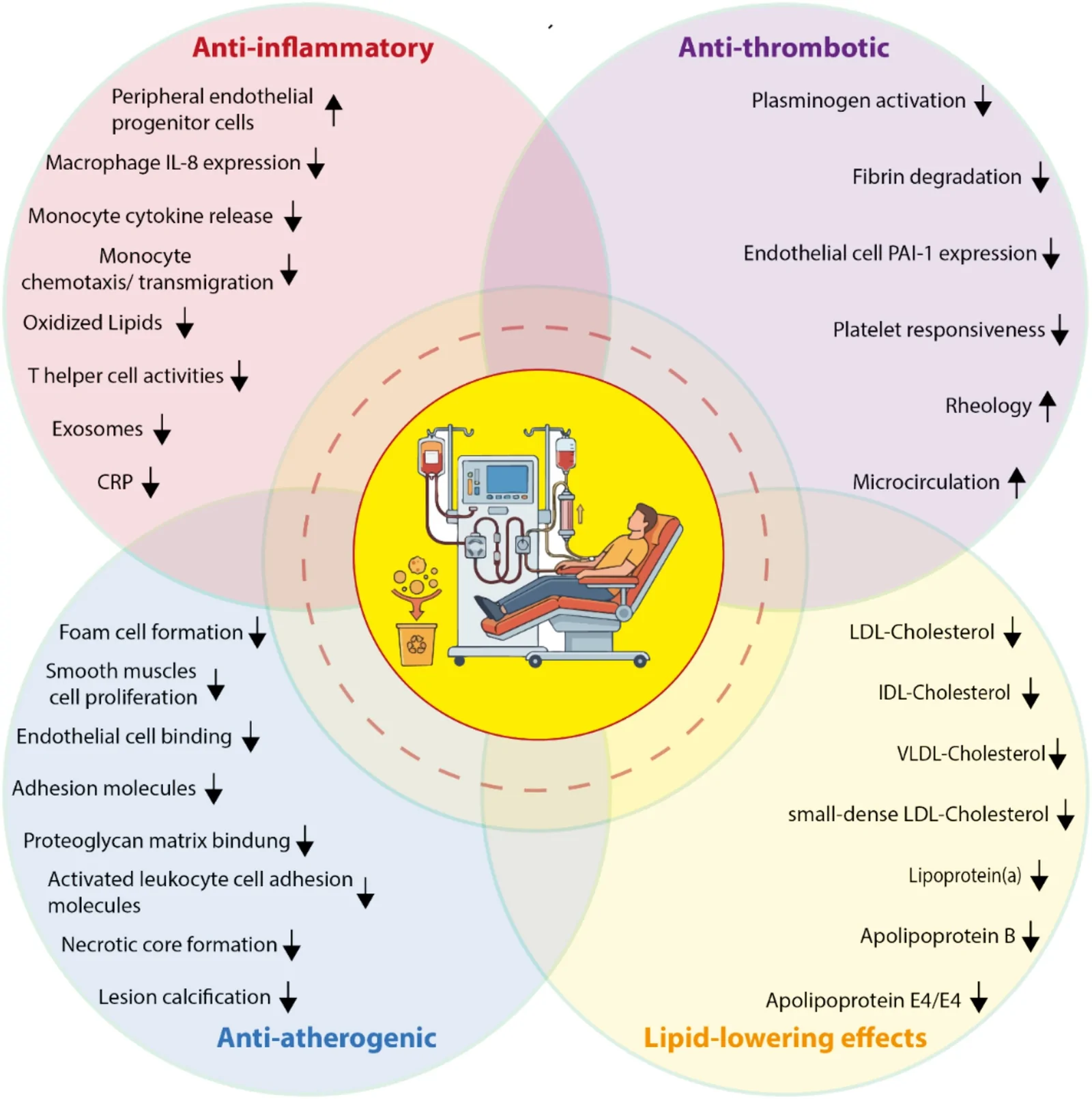
Multimodal (pleiotropic) effects of lipoprotein apheresis during one treatment session.

From the PRO perspective, LA continues to occupy a distinct position in preventive cardiology. Despite major pharmacological advances, there remain clearly defined groups of patients for whom LA is not a relic of the past but an indispensable component of modern cardiovascular risk management (Figure 1).

The first and most paradigmatic indication is HoFH. In HoFH, lifelong exposure to extremely elevated LDL-C drives an aggressive, rapidly progressive form of atherosclerotic disease. LDL-C concentrations above 500 mg/dl are common, and myocardial infarction, ischaemic stroke, and aortic valve stenosis may occur in childhood or early adult life [3]. In many of these patients, LA is instituted at a very young age, sometimes from ∼5 years onwards, to reduce cumulative LDL-C exposure and delay the onset of overt complications [3]. German ambulatory care data indicate that 120 patients with HoFH were receiving LA, predominantly in a primary prevention setting, although this likely underestimates the true burden because centralized capture of all inpatient and semi-inpatient treatments is lacking [6].

A second major indication is severe hypercholesterolaemia in patients who remain inadequately controlled despite contemporary lipid-lowering therapy [1, 2]. Achieving recommended LDL-C targets, particularly the secondary-prevention goal of <55 mg/dl, remains difficult in routine practice, even with intensive combination therapy [1, 2]. Statin intolerance is an important contributor. A clinically relevant subset of patients develops genuine statin-associated myopathy, and autoimmune necrotizing myopathy associated with anti-HMG-CoA reductase antibodies, though rare, is well recognized [1, 2]. Even after all evidence-based options have been exhausted, many patients continue to exhibit substantial residual risk [6].

A third and increasingly prominent indication is isolated elevation of Lp(a). Lp(a) is now firmly established as an independent and likely causal risk factor for ASCVD, with risk rising progressively from about 50 mg/dl, corresponding to ∼105 nmol/l [7]. In patients with markedly elevated Lp(a), otherwise well-controlled LDL-C, and evidence of progressive cardiovascular disease despite rigorous treatment of all modifiable risk factors, LA is regarded as the ultimate therapeutic option [2]. At present, it is the only routinely available intervention in German clinical practice that directly and effectively lowers Lp(a) [2]. According to national data, 2783 patients with isolated Lp(a) elevation received LA [6]. Findings from the German Lipoprotein Apheresis Registry and from the Pro(a)LiFe study suggest that regular LA is associated with pronounced reductions in subsequent cardiovascular events in this highly selected population [8, 9].

The PRO position recognizes that the persistence of these indications does not reflect stagnation in lipid-lowering pharmacology. On the contrary, lipidology is entering a phase of molecular precision medicine. Antisense oligonucleotides and siRNA-based agents can selectively suppress hepatic synthesis of target proteins with remarkable potency and durability. Inclisiran, an siRNA directed against PCSK9 mRNA, is the most mature example for LDL-C lowering. By suppressing hepatic PCSK9 synthesis and enhancing LDL receptor recycling, inclisiran produces sustained LDL-C reductions of around 50% when given at baseline, at 3 months, and then every 6 months [10, 11]. Tolerability has been generally favourable, with injection-site reactions as the most frequent adverse event. However, it remains uncertain whether the degree of LDL-C lowering with inclisiran will translate into the same reduction in hard cardiovascular outcomes as that demonstrated for statins and monoclonal PCSK9 inhibitors.

Gene-silencing approaches targeting Lp(a) have generated even greater interest. Pelacarsen, an antisense oligonucleotide targeting LPA, has produced sustained reductions in Lp(a) exceeding 70% in patients with established coronary artery disease undergoing LA, and may be potent enough to decrease the apheresis burden in that setting [12]. Olpasiran, an siRNA targeting apo(a), has achieved Lp(a) reductions of up to 98% in randomized studies [10]. Other agents, such as zerlasiran and lepodisiran, have also shown profound and durable Lp(a) lowering in early clinical development. Yet the key question remains unanswered: whether these biomarker changes will translate into fewer myocardial infarctions, strokes, and cardiovascular deaths [12].

The PRO argument stresses that, while nucleic acid-based therapies offer exceptional target specificity [10, 12], sustained lipid lowering, and infrequent dosing that may enhance adherence, they are not without limitations. All currently available agents require structured follow-up and subcutaneous administration. Injection-site reactions are common, and there are class-specific safety considerations, including immune stimulation, complement activation, hepatotoxicity, nephrotoxicity, thrombocytopaenia, and potential off-target effects [10]. Their pharmacological durability is achieved through extensive chemical modification of nucleobases, sugar moieties, and phosphodiester backbones, thereby conferring nuclease resistance and prolonging tissue persistence [13]. This design underlies their long-lasting sequence-specific activity, but also means that long-term metabolic handling of these highly modified molecules is not fully characterized. While there is no current evidence that incomplete degradation or incorporation of modified fragments into endogenous nucleic acids is harmful, reversibility is inherently limited: siRNA effects that persist for months are welcome when treatment is well tolerated but can be problematic if serious adverse effects emerge [14].

In this context, the PRO position highlights the distinctive value of LA as an extracorporeal debulking therapy. LA rapidly removes circulating atherogenic lipoproteins, notably apolipoprotein B-containing particles such as LDL and, depending on the method employed, Lp(a). Its effect is immediate and pronounced: over a single treatment session, typically completed within 2–3 h, LDL-C and Lp(a) can be substantially reduced [2]. Beyond this quantitative effect, LA may exert pleiotropic biological actions. By extracting oxidized lipoproteins and other particularly atherogenic particles and by modulating inflammatory mediators, rheological properties, and, in some systems, prothrombotic plasma constituents, LA can act on several axes of atherosclerotic pathobiology simultaneously [1, 2]. These multimodal effects are consistent with observations of improved endothelial function and possible plaque stabilization, and may be especially valuable in patients with refractory angina and elevated Lp(a), in whom improvements in myocardial perfusion, atheroma burden, exercise capacity, and symptom burden have been reported. In very-high-risk populations, registry data and observational studies indicate that regular LA can attenuate or halt the progression of atherosclerotic disease and reduce cardiovascular event rates [8, 9].

For the PRO author, nucleic acid-based therapies and LA should therefore be seen as complementary rather than competing options within an individualized, hierarchical treatment algorithm.

Gene-silencing therapies embody molecular precision medicine but, by design, target single molecular determinants within a broader atherothrombotic process and do not provide comprehensive attenuation of total atherogenic burden, particularly its chronic inflammatory component [15]. The persistence of cardiovascular events despite marked reductions in LDL-C or Lp(a) levels illustrates the reality of residual risk [15]. LA, in contrast, is a multimodal procedural intervention whose role is especially compelling when cardiovascular risk is extreme, when disease progresses despite maximally tolerated conservative therapy, when pharmacological options are constrained by intolerance or contraindication, or when rapid reduction of circulating atherogenic lipoproteins is needed in clinically unstable phases.

Looking ahead, the PRO perspective emphasizes that atherosclerosis should be understood not merely as a disorder of lipoprotein concentration but as a complex inflammatory and atherothrombotic syndrome [15]. This complexity may explain why a multimodal intervention such as LA can continue to provide benefit in selected patients, even against a background of sophisticated pharmacotherapy. The hypothesis that very intensive short-term reduction of LDL-C and/or Lp(a) immediately after acute coronary syndromes may induce a durable legacy effect is also noted as an area of interest, although it remains to be tested formally [2]. Until targeted therapies demonstrate unequivocal long-term outcome benefit, broad accessibility, and the ability to reproduce not only the lipid-lowering efficacy but also the immediacy and potential pleiotropic effects of extracorporeal lipoprotein removal, the PRO position concludes that LA will remain an essential ultima ratio therapy for a subset of patients at the highest end of cardiovascular risk.

## CON POSITION: LIPID APHERESIS IS SOON A HISTORICAL PRACTICE

The CON position starts from the recognition that LA was an innovation born of necessity. In the pre-statin era, and long before PCSK9 inhibition was conceivable, patients with HoFH faced LDL-C levels often exceeding 500 mg/dl and near-certain premature cardiovascular death, commonly in the second or third decade of life. In that context, apheresis offered the only meaningful reduction in atherogenic lipoproteins, and its adoption was entirely rational. The present debate, however, is not about the justification of apheresis in the 1980s, but about its justification in 2025 and beyond, in a pharmacological environment that has changed almost beyond recognition (Table 1).

**Table 1:** Comparison of lipoprotein apheresis and emerging pharmacological alternatives.

Feature	Lipoprotein apheresis	PCSK9 inhibitors/inclisiran	Targeted Lp(a) agents (pelacarsen/olpasiran)	Evinacumab (HoFH)
Route	IV/extracorporeal, weekly to biweekly	SC, monthly or biannually	SC, monthly to quarterly	IV infusion, monthly
LDL-C reduction	65%–85% per session; ∼40%–50% time-averaged	50%–60% sustained	20%–30% (indirect)	47%–49% (HoFH)
Lp(a) reduction	60%–75% per session; ∼25%–40% time-averaged	20%–30% (variable)	80%–97% sustained	Not primary target
RCT outcomes data	None for CVD endpoint vs. modern pharmacotherapy	Yes (FOURIER, ODYSSEY OUTCOMES)	Phase 3 ongoing (results 2025–26)	Limited (FH registries)
Access	∼33 US states; limited globally	Widely available	Pending approval; trials ongoing	Specialty centres
Annual cost (USD)	$50 000–$150 000	$6 000–$14 000 (branded)	TBD (estimated comparable to PCSK9i)	$450 000+
Procedural burden	High: 3–6 h sessions, weekly/biweekly, lifelong	Low: self-injectable	Low: quarterly or biannual injection	Low: monthly infusion
Applies to HoFH	Yes (historically first-line)	Partial (limited LDLr activity)	No (no LDL effect)	Yes (LDLr-independent)

HoFH, homozygous familial hypercholesterolaemia; IV, intravenous; SC, subcutaneous; TBD, to be determined. Costs are approximate US figures.

According to the CON author, the clinical population for which LA was originally approved—patients with HeFH unable to achieve LDL-C targets on maximally tolerated statins—has been profoundly reshaped by modern pharmacology. PCSK9 monoclonal antibodies such as evolocumab and alirocumab reduce LDL-C by about 50%–60% on top of background therapy and have demonstrated reductions in hard cardiovascular outcomes in landmark randomized controlled trials [16]. Inclisiran, an siRNA that silences hepatic PCSK9 synthesis, achieves comparable LDL-C reductions with twice-yearly subcutaneous dosing, offering significant advantages in adherence and scalability [17]. Bempedoic acid provides a statin-independent oral alternative for statin-intolerant patients, and evinacumab, targeting angiopoietin-like 3 (ANGPTL3), is effective even in HoFH because its mechanism does not depend on LDL receptor activity [18]. In practice, this means that the pool of patients who have genuinely exhausted all pharmacological options and truly require LA has been sharply reduced. Many patients currently referred for apheresis either have not received PCSK9 inhibitors, have not reached adequate doses, or are potential candidates for newer agents that have not been offered by their treating clinicians. Referral pathways to apheresis, in many health systems, have not kept pace with the pharmacological pipeline.

A central plank of the CON argument is the weak evidentiary basis underpinning claims of cardiovascular benefit from LA. Proponents often quote reductions in major adverse cardiovascular events of 50%–85% in published series. The entire cardiovascular outcomes literature on LA, however, consists almost exclusively of uncontrolled observational studies that compare event rates in the same patients before and after apheresis initiation, using individuals as their own historical controls [6]. This design is prone to regression to the mean, temporal confounding, intensification of concomitant therapy, and lifestyle changes that accompany program enrolment. As a result, it is difficult to ascribe observed reductions in events specifically to LA rather than to the comprehensive optimization of pharmacological therapy and risk factor management occurring in parallel. No adequately powered randomized controlled trial has demonstrated that LA reduces cardiovascular events compared with contemporary, maximally optimized pharmacological therapy. For the CON author, this is not a minor methodological footnote but the central evidentiary gap of the entire field. The 2024 American Heart Association Scientific Statement on LA explicitly acknowledged this gap and called for randomized controlled trials or large prospective studies to substantiate cardiovascular benefits [19].

Isolated Lp(a) hyperlipoproteinaemia is recognized in both manuscripts as the last stronghold of LA. Statins, PCSK9 inhibitors, and ezetimibe have modest or negligible effects on Lp(a) [12]. LA can acutely reduce Lp(a) by roughly 60%–75% per treatment, but inter-session rebound means that the time-averaged reduction from baseline is nearer to 25%–40% [20]. The supporting evidence here is also largely observational and registry-based [21]. From the CON perspective, this niche is already being filled by pharmacology. Pelacarsen, an antisense oligonucleotide targeting apo(a) mRNA, achieves mean reductions in Lp(a) of about 80%, with 98% of subjects in phase 2 trials reaching desirable Lp(a) concentrations [22]. Olpasiran, an siRNA agent, has produced placebo-adjusted reductions above 97% at higher doses in the OCEAN(a)-DOSE trial [23]. Lepodisiran, zerlasiran, and the oral agent muvalaplin offer further mechanistic diversity. Large phase 3 cardiovascular outcome trials, including Lp(a)HORIZON for pelacarsen (with more than 8300 participants) and OCEAN(a) for olpasiran (with over 7000 participants), are expected to report between 2025 and 2026 [24]. If these agents are approved, they will provide subcutaneous therapies, administered monthly or even less frequently, that target Lp(a) specifically and avoid the vascular access demands and time commitments of apheresis. For the CON author, the rational response to this evolving landscape is to ask whether patients currently being enrolled in apheresis programs for elevated Lp(a) might instead be better served by inclusion in ongoing outcome trials or access programs for targeted Lp(a)-lowering agents. In this view, the period during which apheresis is the only option for Lp(a) reduction is drawing to a close.

### Beyond efficacy, the CON position underlines the procedural and life-course burdens of LA

Regular LA treatment requires reliable large-bore vascular access, usually through peripheral veins, an arteriovenous fistula, or a tunnelled central venous catheter. Over time, peripheral access often fails, particularly in elderly patients or those with small or fragile veins, leading to fistula surgery or long-term central venous catheterization, with attendant risks of infection, thrombosis, and central vein stenosis that accumulate over decades of therapy. The commitment is essentially lifelong, since LA functions as a permanent replacement for a failing biochemical pathway. Unlike tablets or injections that can be paused or discontinued, apheresis requires patients to structure their work, travel, and family life around regular sessions. Surveys of patients on long-term apheresis consistently report treatment burden as a major concern, and discontinuation rates rise as patients age and their comorbidity profile changes. For a young person with HoFH, initiating apheresis at 10 or 15 years of age implies a major lifelong commitment that, in the CON view, must be weighed against the trajectory of pharmacological alternatives rather than accepted as the default.

Cost and equity considerations further reinforce this argument. Annual costs for LA in the United States are estimated at 50 000–150 000 US dollars, depending on the care setting and payer, and do not account for indirect costs related to lost work, travel, and caregiver time [4]. Globally, LA is largely restricted to high-income countries and wealthier segments of the population. As PCSK9 inhibitor prices fall and long-acting Lp(a)-lowering agents approach the market, the cost-effectiveness of operating and expanding apheresis programs becomes increasingly difficult to defend.

The CON author acknowledges that the current indication for apheresis is not literally zero. Patients with true HoFH who harbour two null LDL receptor mutations derive little or no benefit from PCSK9 inhibitors, which require residual LDL receptor activity. For these individuals, evinacumab, lomitapide, and apheresis remain options of last resort. HoFH is rare, affecting ∼1 in 300 000 individuals worldwide, but for these patients, treatment options remain limited. Similarly, a small subset of patients with isolated Lp(a) hyperlipoproteinaemia and recurrent cardiovascular events, despite exhaustive lipid-lowering therapy, may justifiably receive apheresis as a bridging intervention until targeted Lp(a)-lowering agents are available. The CON perspective, however, challenges the extrapolation of this narrow, authentic indication to much larger populations with HeFH or elevated Lp(a) who have not been systematically optimized on modern pharmacological therapy.

In nephrology, LA has also been investigated in focal segmental glomerulosclerosis (FSGS) and refractory nephrotic syndrome. The mechanistic rationale is plausible: LDL apheresis may remove circulating permeability factors, reduce oxidized LDL-mediated podocyte damage, and attenuate inflammatory mediators involved in glomerular injury. Nevertheless, the results have been inconsistent. Published series are small, uncontrolled, and heterogeneous in patient selection, apheresis modality, concomitant immunosuppression, and outcome definitions. A 2025 comprehensive review of apheresis in kidney diseases reported improvements in selected patients but highlighted the absence of randomized comparators and the difficulty in differentiating treatment effects from spontaneous partial remissions that occur in some FSGS subtypes [25]. For the CON author, these nephrology indications epitomize the broader apheresis literature: mechanistically appealing hypotheses and promising single-centre reports, but a persistent failure to generate the robust controlled data necessary for confident clinical recommendations.

The CON conclusion is that LA served an important purpose when effective drug options were lacking, but that the era in which LA is widely necessary is ending. PCSK9 inhibitors and inclisiran have largely eliminated the HeFH indication for patients who are appropriately treated pharmacologically. Emerging Lp(a)-targeted siRNA and antisense therapies are on the verge of eliminating the Lp(a) apheresis niche. The residual population that genuinely requires LA, principally null-mutation HoFH patients who have exhausted evinacumab, lomitapide and other available options, is very small and does not justify maintaining a broad, resource-intensive apheresis infrastructure at scale, particularly given the continuing lack of randomized outcomes data and the substantial burden, costs, and inequities associated with LA.

## MODERATORS' CONCLUSION

When the two positions are considered together, several important points of agreement and divergence emerge. Both authors recognize that HoFH and other extreme lipid phenotypes represent some of the highest-risk states in cardiovascular medicine and that, in these settings, timely and aggressive intervention is crucial. Both agree that lipoprotein apheresis can rapidly and substantially reduce LDL-C and Lp(a), and that observational data and registry analyses suggest clinical benefits in highly selected, very high-risk patients. Both acknowledge that the pharmacological environment has been transformed by high-potency LDL-C-lowering agents and by the arrival of highly effective, target-specific Lp(a)-lowering antisense and siRNA therapies. Finally, both accept that the evidence base for lipoprotein apheresis is limited by the absence of adequately powered randomized, controlled outcome trials, and that leading scientific statements now explicitly call for more rigorous evaluation.

The main difference lies in how these shared facts are interpreted. The PRO author emphasizes that, despite therapeutic innovation, there remains a significant minority of patients whose risk is not sufficiently controlled by even the most advanced pharmacological regimens, and for whom lipoprotein apheresis provides a unique combination of rapid, multimodal and reversible lipoprotein debulking. In this view, lipoprotein apheresis functions as an essential ultima ratio therapy and a bridge in an evolving therapeutic landscape. The CON author, by contrast, stresses that the true number of patients who cannot be adequately treated with contemporary pharmacology is already small and likely to diminish further as RNA-based agents gain outcome data and regulatory approval. Given the burdens, complications, costs, and inequities in access associated with lipoprotein apheresis, and the lack of randomized outcome trials, this view sees lipoprotein apheresis as a technology that should recede into a narrowly defined niche and eventually into history, rather than remain a broadly deployed chronic treatment.

A balanced position integrates elements of both. In the present moment, lipoprotein apheresis appears to retain a legitimate, if narrow, role in the care of patients at the extreme end of lipid-driven risk: those with null-mutation HoFH or comparable phenotypes in whom state-of-the-art pharmacotherapy, including PCSK9 inhibition and, where available, evinacumab and lomitapide, has been fully exploited yet remains insufficient; and those with isolated, very high Lp(a) and convincingly progressive atherosclerotic cardiovascular disease who cannot access or are ineligible for Lp(a)-targeted clinical trials or programs. Even in these groups, lipoprotein apheresis should generally be considered a bridging strategy and should be offered only after careful documentation that all appropriate pharmacological options have been used or are contraindicated.

At the same time, the threshold for initiating lipoprotein apheresis should continue to rise as new pharmacological agents become available and as high-quality outcome data clarify their impact on hard endpoints. Referral pathways and guidelines should be updated to ensure that patients undergo systematic pharmacological optimization before apheresis is contemplated. Wherever feasible, the comparative effectiveness of lipoprotein apheresis versus modern, fully optimized drug therapy should be examined in prospective, controlled studies, particularly in key niches where lipoprotein apheresis is still expected to be used. In parallel, lipoprotein apheresis services can contribute importantly by facilitating -the identification and characterization of extreme-risk patients, thereby supporting recruitment and phenotyping in ongoing and future trials of RNA-based and other novel agents.

Viewed in this way, lipoprotein apheresis can be seen neither as a therapy that should be abandoned precipitously nor as one that ought to be preserved unchanged. Rather, it is a modality whose field of application is likely to contract over time as targeted pharmacological therapies prove their efficacy, longterm safety, and cost efficacy and, become more widely available. Over the coming decade, if indeed Lp(a)-directed and other advanced therapies demonstrate robust cardiovascular outcome benefit and if their access and affordability improve, LA will probably evolve into a specialized intervention reserved for rare, extreme phenotypes and specific unstable clinical situations, while its once broad role in chronic lipid management will largely become a chapter in the history of cardiovascular prevention.

## References

[1] Nadim M, Akgun Y. Beyond lipids: systemic effects of lipoprotein apheresis. Transfusion. 2026;66:590–8. 10.1111/trf.70083 41538799 PMC12983121

[2] Schettler VJJ . Lipoprotein apheresis: current overview and future outlook in clinical practice. Transfusion Apheresis Sci. 2025;64:104209. 10.1016/j.transci.2025.104209 40700816

[3] Cuchel M, Raal FJ, Hegele RA et al. 2023 Update on European Atherosclerosis Society Consensus Statement on homozygous familial hypercholesterolaemia: new treatments and clinical guidance. Eur Heart J. 2023;44:2277–91. 10.1093/eurheartj/ehad197 37130090 PMC10314327

[4] Schettler VJJ . Lipoprotein apheresis: still needed in many patients. Clin Kidney J. 2026; in press. 10.1093/ckj/sfag240 PMC13492341 42626375

[5] Akgun Y. Lipid apheresis: soon a historical practice (CON). Clin Kidney J. 2026; in press. 10.1093/ckj/sfag240 PMC13492341 42626375

[6] National Association of Statutory Health Insurance Physicians . Quality Report 2025: Reporting Year 2024. Berlin: National Association of Statutory Health Insurance Physicians, 2025.

[7] Mach F, Koskinas KC, Roeters van Lennep JE et al. 2025 focused update of the 2019 ESC/EAS Guidelines for the management of dyslipidaemias. Eur Heart J. 2025;46:4359–78. 10.1093/eurheartj/ehaf190 40878289

[8] Schettler VJJ, Selke N, Jenke S et al. The German lipoprotein apheresis registry: summary of the eleventh annual report. Atherosclerosis. 2024;398:118601. 10.1016/j.atherosclerosis.2024.118601 39342791

[9] Klingel R, Julius U, Bernhardt WM et al. Lipoprotein apheresis for lipoprotein(a)-associated progressive atherosclerotic cardiovascular disease: 12-years follow-up. Atherosclerosis. 2025;410:120508. 10.1016/j.atherosclerosis.2025.120508 41124886

[10] Katzmann JL, Laufs U. Choosing the right non-statin therapy for the right patient: how to sequence advanced lipid-lowering therapies. Curr Atheroscler Rep. 2026;28:1390–7. 10.1007/s11883-026-01390-7 PMC12945982 41746457

[11] Blais JE, Zhong W, Li CYV et al. Adherence to PCSK9 inhibitors in clinical practice: systematic review and meta-analysis of observational studies. JACC Advances. 2026;5:102733. 10.1016/j.jacadv.2026.102733 42000553 PMC13098587

[12] Nordestgaard BG, Langsted A. Lipoprotein(a) and cardiovascular disease. Lancet. 2024;404:1255–64. 10.1016/S0140-6736(24)01308-4 39278229

[13] Yu RZ, Graham MJ, Post N et al. Disposition and pharmacology of a GalNAc3-conjugated antisense oligonucleotide targeting human lipoprotein(a) in mice. Mol Ther Nucl Acids. 2016;5:e317. 10.1038/mtna.2016.26 PMC5014512 27138177

[14] Landmesser U, Poller W, Tsimikas S et al. From traditional pharmacological towards nucleic acid-based therapies for cardiovascular diseases. Eur Heart J. 2020;41:3884–99. 10.1093/eurheartj/ehaa229 32350510

[15] Sallam T, van Solingen C, Moore KJ. Non-coding RNAs in lipid metabolism and their roles in atherosclerosis. Nat Rev Cardiol. 2026;23:324–45. 10.1038/s41569-025-01229-9 41478885 PMC13525632

[16] Sabatine MS, Giugliano RP, Keech AC et al. Evolocumab and clinical outcomes in patients with cardiovascular disease. N Engl J Med. 2017;376:1713–22. 10.1056/NEJMoa1615664 28304224

[17] Raal FJ, Kallend D, Ray KK et al. Inclisiran for the treatment of heterozygous familial hypercholesterolemia. N Engl J Med. 2020;382:1520–30. 10.1056/NEJMoa1913805 32197277

[18] Raal FJ, Rosenson RS, Reeskamp LF et al. Evinacumab for homozygous familial hypercholesterolemia. N Engl J Med. 2020;383:711–20. 10.1056/NEJMoa2004215 32813947

[19] Gianos E, Duell PB, Toth PP et al. Lipoprotein apheresis: utility, outcomes, and implementation in clinical practice: a scientific statement from the American Heart Association. Arteriosclerosis Thrombosis Vasc Biol. 2024;44:1–22. 10.1161/ATV.0000000000000177 39370995

[20] Thompson GR . The scientific basis and future of lipoprotein apheresis. Ther Apher Dial. 2022;26:32–36. 10.1111/1744-9987.13716 34331508

[21] Leebmann J, Roeseler E, Julius U et al. Lipoprotein apheresis in patients with maximally tolerated lipid-lowering therapy, lipoprotein(a)-hyperlipoproteinemia, and progressive cardiovascular disease. Circulation. 2013;128:2567–76. 10.1161/CIRCULATIONAHA.113.002432 24056686

[22] Tsimikas S, Karwatowska-Prokopczuk E, Gouni-Berthold I et al. Lipoprotein(a) reduction in persons with cardiovascular disease. N Engl J Med. 2020;382:244–55. 10.1056/NEJMoa1905239 31893580

[23] O’Donoghue ML, Rosenson RS, Gencer B et al. Small interfering RNA to reduce lipoprotein(a) in cardiovascular disease. N Engl J Med. 2022;387:1855–64. 36342163 10.1056/NEJMoa2211023

[24] Sosnowska B, Toth PP, Razavi AC et al. 2024: the year in cardiovascular disease, the year of lipoprotein(a). Arch Med Sci. 2025;21:355–73. 40395905 10.5114/aoms/202213 PMC12087327

[25] Raina R, Chan EY-H, Hu J et al. Indications and recent evidence for apheresis in children and adults with kidney diseases: a comprehensive review. Clin Kidney J. 2025;18:1–15. 10.1093/ckj/sfaf282 PMC12511939 41079156

